# Immunoglobulins as Biomarkers for Gastrointestinal Nematodes Resistance in Small Ruminants: A systematic review

**DOI:** 10.1038/s41598-020-64775-x

**Published:** 2020-05-08

**Authors:** H. M. Aboshady, M. J. Stear, A. Johansson, E. Jonas, J. C. Bambou

**Affiliations:** 1 AgroParisTech, Paris, France; 2 Department of Animal Breeding and Genetics, Swedish University of Agriculture Science, Uppsala, Sweden; 3 URZ, Unité de Recherches Zootechniques, INRA, 97170 Petit Bourg, (Guadeloupe) France; 4 Department of animal production, Faculty of Agriculture, Cairo University, Cairo, Egypt; 5 La Trobe Univ, Dept Anim Plant & Soil Sci, AgriBio, Ctr AgriBiosci, Melbourne, Vic Australia

**Keywords:** Immunogenetics, Animal breeding

## Abstract

The rise of anthelmintic resistance worldwide has led to the development of alternative control strategies for gastrointestinal nematodes (GIN) infections, which are one of the main constraints on the health of grazing small ruminants. Presently, breeding schemes rely mainly on fecal egg count (FEC) measurements on infected animals which are time-consuming and requires expertise in parasitology. Identifying and understanding the role of immunoglobulins in the mechanisms of resistance could provide a more efficient and sustainable method of identifying nematode-resistant animals for selection. In this study we review the findings on immunoglobulin response to GIN in the literature published to date (june 2019) and discuss the potential to use immunoglobulins as biomarkers. The literature review revealed 41 studies which measured at least one immunoglobulin: 35 focused on lamb immune response (18 used non-naïve lambs) and 7 on yearlings. In this review we propose a conceptual model summarizing the role of immunoglobulins in resistance to GIN. We highlight the need for more carefully designed and documented studies to allow comparisons across different populations on the immunoglobulin response to GIN infection.

## Introduction

Small ruminants are an important source of food and revenue^1,2^. The world’s sheep and goat populations have increased steadily over the past decades, especially in developing countries^2^. One of the main constraints on small ruminant production is management of animal health. Infection with gastrointestinal nematode parasites has the greatest impact upon animal health and productivity^3^. The control of GIN in sheep and goats has been dependent on the use of anthelmintic treatment, however their extensive use has resulted in the anthelmintic resistance^4–6^ which has been reported in multiple countries^7^. In addition, there is a growing demand from consumers to produce chemical-free food and increasing concern about animal welfare^8^.

Therefore, two main axes of research have been identified to develop alternative control strategies for GIN management. The first option is the reduction of parasite burden on the pasture through grazing management. However, nematode-free pastures are not readily available under intensive grazing conditions. The second option to reduce GIN infections is the improvement of the host immune response through the genetic selection of lines or breeds of resistant animals, nutritional supplementation and/or vaccination.

A number of studies have already identified sheep breeds, such as the Florida Native^9,10^, Santa Ines^11,12^, Texel^13,14^, St. Croix^9,15,16^ and Red Massai sheep^17^ that are resistant to various GIN species. There are also reports on differences between breeds in resistance to GIN infection in goats^18–20^. Moreover, variation among individuals within the same breed in response to GIN infection has been observed in sheep^21^ and goats^22,23^, which could be used to breed resistant lines for several breeds. These variations were often applied to breed diverse lines in experimental studies for the identification of mechanisms or genetic regions for GIN resistance.

Several studies have indicated that genetic resistance to GIN is associated with a protective immune response which is mediated, at least partly, by the humoral response^24^. Understanding the differences in the humoral response between resistant and susceptible breeds, lines or individuals could help to design and implement appropriate control programs and sustainable breeding for GIN resistance. To our knowledge, there is no recent review on the association of immunoglobulin responses and the intensity of GIN infection (based on FEC and/or parasite burden counts). The role of different immunoglobulins in immunity to nematodes needs to be confirmed. The objective of this study was to evaluate the role of immunoglobulin responses (especially IgA and IgE) against GIN and their potential use as biomarkers in breeding schemes.

## Results and Discussion

### Evaluation of the risk of bias

**Figure 1:**
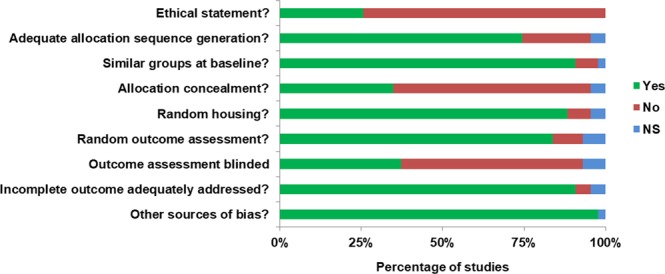
Qualitative evaluation of the risk of bias for the studies included in the systematic review. *Footnotes: Yes: Percentage of studies scoring with low risk of bias. No: Percentage of studies scoring with high risk of bias risk of bias. NS: Percentage of studies that did not specify the key methodological variables*.

**Figure 2:**
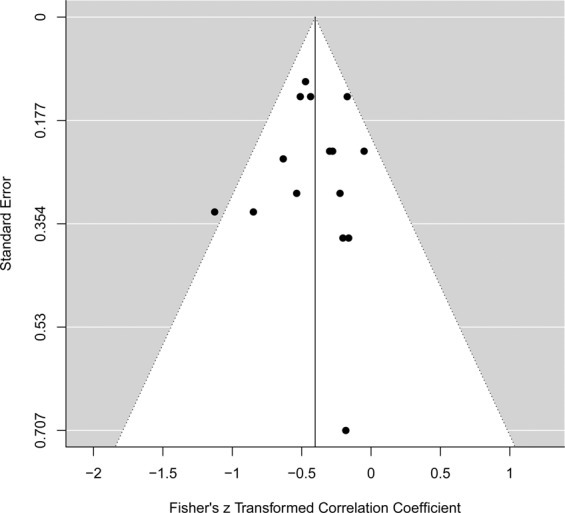
Funnel plot of studies reporting correlation coefficients.

The results of the qualitative evaluation of the risk of bias are presented on Fig. 1. Ethical statement for the use of animal was specified in 26% of the studies. Indeed, this statement was not compulsory before the early 2000s, depending on the country. An adequate allocation sequence generation was specified in 74% of the studies, 88% used random housing and 91% reported the use of similar groups at baseline. However, allocation concealment was not performed in 60% of the studies. Random outcome assessment was specified in 84% of the studies but the outcome assessment was not blind in 56% of the studies. Incomplete outcome was adequately addressed in 91% of the studies and other sources of bias were reported in 98% of the studies. There was no indication of bias across studies reporting correlation coefficients based on the funnel plot (Fig. 2) analysis and the results of the Egger’s test (I^2^ = 10.1%, *P* = 0.304).

### Immune response to GIN infection

Both cellular and humoral responses are actively involved in immune response against nematode infection. The main effectors of this immune response are T- and B-lymphocytes, plasma cells, mast cells, eosinophils, globule leukocytes, soluble cytokines and various immunoglobulin isotypes^25^. Incoming nematode larvae from GIN infection trigger local inflammation and mast cell degranulation, which damages the gastrointestinal mucosa^26^. Dendritic cells, macrophages and the other antigen-presenting cells, capture the nematode antigens within the intestinal mucosa and migrate to the regional lymph nodes to present these antigens to naïves T cells^24^. After T-cell differentiation, the secretion of type 1 T helper (Th1) or type 2 T helper (Th2)-associated cytokines induces the migration to the site of infection of activated effector cells such as eosinophils and mast cells^24,27^. The type of helper CD4 + T lymphocytes that develop following an infection with nematodes is critical for the ability of the host animal to overcome an infection^28^. The Th1 cells produce interferon-gamma (IFNγ), interleukin (IL-2) and tumor necrosis factor-beta (TNFβ) for the activation of macrophages and the initiation of the cell-mediated immunity and the phagocyte-dependent protective responses^11,29^. The Th1 cells develop mainly following infections by intracellular parasites (viruses and some bacteria). When GIN antigens penetrate the gastrointestinal tissues of the host, macrophages and other cells which have receptors for nematode cell surface molecules are activated and induce a specific but mostly ineffective immune response^29^.

The phagocyte-independent protective responses characterized by antibodies production, eosinophils activation and inhibition of several macrophage functions, are activated by the production of Th2 cytokines (IL-4, IL-5, IL-10, and IL-13)^11,29^. The Th1 response inhibits the Th2 response through IL-10^30^, which makes Th1 and Th2 responses antagonistic to each other. Results from studies in sheep showed that CD4 + lymphocytes increase during the experimental infection of both susceptible and resistant sheep with GIN^31,32^. But compared with resistant sheep, susceptible produce more interferon gamma (IFNγ), fewer parasite-specific serum antibodies, blood and abomasal eosinophils^33^. The role of the two major types of T helper cells distinguishes resistant from susceptible sheep.

The acquired immune response after infection with *Haemonchus contortus* was compared in Barbados Blackbelly sheep, which are generally defined as a resistant breed, and Columbia sheep, a breed classified as susceptible^34^. Sheep of the resistant breed developed and sustained a Th2 response through increasing and maintaining IgG and blood eosinophil levels. Meanwhile sheep of the susceptible breed showed changes in the response starting with an initial increase in IgG and blood eosinophils (Th2) but a later reduction in both, which suggests a switch to a Th1 response^34^. An earlier study suggested that in the relative absence of Th1 type secretions (i.e. cytokines), the Th2 cells secrete cytokines that promote mastocytosis, eosinophilia and the production of IgE and IgG1^35^. Gulf coast native (resistant) lambs showed a significantly higher expression of IL-4 mRNA (Th2) on day 10 post exposure to the nematode compared to Suffolk lambs (susceptible). On the other hand, the expression of IFN-γ mRNA and IL-10 (Th1 and regulatory T) on days 7, 10 and 14 post exposure was higher in Suffolk lambs (susceptible) compared with native lambs^25^.

This confirms that if T helper cells of the Th2 type gain ascendancy after GIN infection, then a protective immune response ensues. In contrast, if an inappropriate Th1 type response predominates, effective resistance is unlikely to develop. The Th1 type response for GIN infection is most likely associated with susceptibility, while a Th2 type response is associated with resistant phenotypes in sheep^25,33,34^. As Th2-associated cytokines target plasma cells to produce nematode-specific antibodies and generate protective immune responses^24^, we focus here on the immunoglobulin response against GIN.

### Immunoglobulin response in sheep (IgA, IgE, IgG and IgM)

The association between different immunoglobulin isotypes, including IgA, IgE, IgG and IgM and GIN resistance has been widely studied in sheep (Table 1). Studies which measured at least one immunoglobulin parameter during GIN infection (Table 1) differed in sheep breed used, type of breed (resistant or susceptible), age of animals in the experiment, immunological status (naïve or non-naïve), infected parasite genus and infection type (natural, artificial with single dose or artificial with trickle doses) which makes the comparison between them rather complex.

**Table 1:** Studies in sheep with at least one immunoglobulin against gastrointestinal nematode measured.

Breed	Gen^a^	n^b^	Age Class	Immune status	GIN sp.^c^	Inf.^d^	Reference
Texel	R	6	lamb	non-naïve	*T. circumcincta*	AIs	^94^
Suffolk	S	5					
Santa Ines	R	16	yearling	non-naïve	*H. contortus*	NI	^11^
Ile de France	S	9					
Suffolk	S	11					
Santa Ines		15	yearling	naïve	*H. contortus*	NI	^55^
Dorper x Santa Ines		15					
Ile de France x Santa Ines	R	15					
Suffolk × Santa Ines		15					
Texel × Santa Ines		15					
Merino	R&S	14	lamb	naïve & non-naïve	*H. contortus & T. colubriformis*	AIs	^61^
Blackface	R&S	57	lamb	naïve	*T. circumcincta*	AIt	^42^
Romney	R&S	65	lamb	non-naïve	natural mixed	NI	^38^
St. Croix hair	R	10	lamb	non-naïve	*H. contortus*	AIs	^16^
Wool^e^	S	10					
St. Croix hair	R	6	lamb	non-naïve	*H. contortus*	AIs	^95^
Wool^e^	S	6					
Santa Ines		20	lamb	non-naïve	*T. colubriformis*	AIt	^56^
Manchego	—	9	lamb	naïve & non-naïve	*H. contortus*	AIs	^96^
Scottish Blackface	—	1000	lamb	non-naïve	natural mixed	NI	^47^
Romney	—	10	lamb	naïve	*T. colubriformis*	AIt	^54^
Romney	R&S lines	1547	lamb	non-naïve	*T. colubriformis*	NI	^36^
Scotch Mule^f^	—	23	lamb	non-naïve	*T. circumcincta*	AIs	^39^
Rhön	S	133	lamb	naïve	*H. contortus*	AIs	^37^
Merinoland	R	244					
Marino		14	lamb	naïve & non-naïve	*H. contortus*	AIs	^97^
Manchego	R	12					
Castellana		13	lamb	naïve & non-naïve	*H. contortus*	AIs	^85^
Churra	—	14	lamb	naïve & non-naïve	*H. contortus*	AIs	^98^
Blackbelly	R	16	lamb	non-naïve	*T. colubriformis*	NI & AIs	^99^
Scottish Blackface-cross	—	46	yearling	naïve & non-naïve	*T. circumcincta*	AIs	^100^
Scottish Blackface		30	lamb	non-naïve	*T. circumcincta*	AIs	^41^
Canaria Hair	R	18	yearling	non-naïve	*H. contortus*	AIs	^101^
Canaria	S	19					
Greyface cross Suffolk	—	28	lamb	naïve & non-naïve	*T. circumcincta*	AIs	^49^
Greyface cross Suffolk	—	28	lamb	naïve & non-naïve	*T. circumcincta*	AIs	^102^
Texel	—	256	lamb	naïve & non-naïve	*H. contortus*	AIs	^103^
INRA 401	—	81	lamb	naïve & non-naïve	*H. contortus*	AIs	^86^
St. Croix hair	R	26	lamb	non-naïve	*H. contortus*	AIs	^15^
Wool^e^	S	26					
Scottish Blackface	R&S	20	lamb	non-naïve	*T. circumcincta*	AIs	^21^
Churra	—	22	yearling	non-naïve	*T. circumcincta*	AIs	^40^
Blackbelly	R	27	lamb	naïve	*H. contortus*	AIs	^34^
Columbia	S	29					
Texel	R	—	lamb	non-naïve	*T. circumcincta*	NI	^82^
Santa Ines crossbred	—	54	lamb	non-naïve	*H. contortus & H. placei*	AIt	^57^
Suffolk	S	57	lamb	naïve	*T. circumcincta*	NI	^13^
Texel	R	85					
Texel	—	256	lamb	naïve & non-naïve	*H. contortus*	AIs	^60^
Gulf Coast Native	R	30	lamb	non-naïve	*H. contortus*	AIst	^33^
Suffolk	S	30					
Romney	R&S lines	816	lamb	non-naïve	*T. colubriformis*	NI	^50^
Romney	R&S lines	21	yearling	naïve & non-naïve	T. colubriformis	AIs	^51^
Romney × Texel × Finnish Landrace	—	614	lamb	non-naïve	*H. contortus*	NI	^45^
Santa Ines	R	10	lamb	non-naïve	*H. contortus & T. colubriformis*	NI	^12^
Ile de France	S	12					
INRA 401	S	28	lamb	naïve & non-naïve	*H. contortus*	AIs	^104^
Barbados Black Belly	R	25					
Marino	R line	20	yearling	non-naïve	*T. colubriformis and/or T. circumcincta*	AIt	^105^
St. Croix	R	20	lamb	naïve & non-naïve	*H. contortus*	AIs	^9^
Florida Native	R	12					
Dorset/Rambouillet	S	16					

^a^Gen.: Genotypes Resistant (R) and Susceptible (S).

^b^n: Number of animals/genotype.

^c^GIN sp.: Gastrointestinal nematode species.

^d^Inf.: Infection protocol, single artificial infection (AIs), trickle artificial infection (AIt), natural infection (NI).

^e^50% Dorset 25% Finnsheep 25% Rambouillet.

^f^Blackface ewe × Blue-faced Leicester ram.

**Figure 3:**
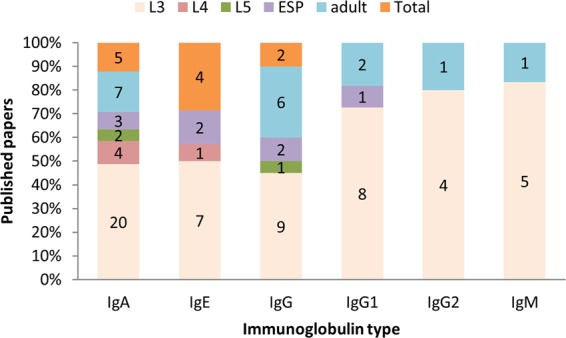
Percentages and numbers of published articles measuring different immunoglobulins levels against different larva stage during GIN infection.

When comparing publications which measured different immunoglobulins levels against different larva stages during GIN infection in sheep it was found that the majority of the studies examined the presence of L3 antigen-specific immunoglobulins (Fig. 3). The third stage larva (L3) represents the stage with the first contact of the gastrointestinal nematodes with the host immune system. It can also be seen from the figure that IgA was the most commonly investigated immunoglobulin isoform in sheep.

### Total antibody response

Results from Douch *et al*.^36^ and Gauly *et al*.^37^ suggested that resistant sheep have higher total antibody levels and that the antibody level could be used in selection for resistance. Romney rams, selected based on their serum antibody levels at an age of 6 months to reduce FEC, underwent a natural parasite challenge, and it was predicted that the genetic gain was 51 to 67% of the genetic gain achieved when FEC was directly used as a selection trait^36^. The total antibody level in both 4 and 5 months old Rhön (resistant) sheep were significantly higher compared to Merinoland (susceptible) lambs following experimental infection with *H. contortus*^37^.

The correlation between *Trichostrongylus colubriformis*-L3 or -adult total antibody and FEC (−0.62 and −0.55) or nematode burden (−0.56 and −0.63) was high in Romney progeny selected for low and high FEC following a natural GIN challenge^38^.

### IgA response

It has been suggested that local immune effectors expressed in the abomasal mucosa, particularly IgA, play an important role in immunity acquired both naturally and experimentally^39^. In sheep, secretory and plasma IgA derive predominantly from the gastrointestinal tract^24^. The correlation between gastric mucus IgA and peripheral IgA is positive and highly significant, ranging from 0.618 to 0.779^40,41^. Several studies reported an increase of the IgA response after GIN infection, higher levels were recorded for resistant breeds^16,39,42^.

The level of IgA against the CarLA antigen (a carbohydrate larval surface antigen expressed on the L3 of all trichostrongylid nematode species) has been suggested to be a suitable means to measure the level of resistance to GIN^43,44^. Different studies found that CarLA is a target antigen for host IgA which binds to the larval surface antigen and prevents larvae from establishing at their preferred sites in the intestinal epithelial folds^43–45^. In this context, a different L3-specific surface antigen (CarLA) was detected from *Trichostrongylus colubriformis, Haemonchus contortus* and *Ostertagia circumcincta* with similar molecular weight (35-kDa), and from *Cooperia curticei* and *Nematodirus spathiger* with a different molecular weight (22-kDa on blots of L3 extracts)^43^. IgA in saliva had a negative genetic correlation with FEC (*r* = −0.5) and animals with high levels of anti-CarLA IgA have typically 20–30% lower FEC than animals with low or undetectable titers^45^. A simple way to use these results for the selection of animals resistant to parasite infection could be to measure anti-CarLA IgA in saliva.

The faecal egg output in St. Croix hair-type sheep (resistant breed) can rapidly reduce in response to *H. contortus* artificial infection following a 45-day breeding season, which was accompanied with higher levels of circulating antigen-specific antibody IgA compared to a susceptible composite line of wool-type sheep (50% Dorset, 25% Finnsheep and 25% Rambouillet breeding)^16^. The increase in anti-*T. circumcincta* IgA antibody and eosinophil concentrations were associated with an increase in the frequency of early L4^42^. Also Ellis *et al*.^39^ found a correlation (*r* = 0.534, *P* = 0.007) between L3 antigen-specific IgA levels in efferent gastric lymph and the percentage of inhibited L4s. In addition, a negative correlation (*r* = −0.565, *P* = 0.005) between total *T. circumcincta* burden measured at necropsy and L3 antigen-specific IgA levels in efferent gastric lymph was reported..

A negative association was reported between IgA activity against L4 and both egg counts and worm length when studying resistance to *T. circumcincta* in Scottish Blackface and Churra sheep^40,46^. Gastric mucus IgA against L4 somatic antigen was highly and negatively correlated (*r* = −0.71, *P* < 0.01) with the number of eggs per female in utero and also with the length of adult females (*r* = −0.552, *P* < 0.01). Results for IgA against somatic antigen from the adult stage were similar to those with activity against L4, but correlations were somewhat weaker^40^. Negative genetic correlations were found between IgA and FEC, worm length, worm fecundity and worm burden (*r* = −0.78, −0.53 and −0.62, −0.36, respectively) in Scottish Blackface lambs exposed to natural mixed infection^47^. It was suggested that parasite development such as worm growth and fecundity in sheep can be regulated via an IgA response, possibly in conjunction with eosinophils^11,41^.

**Figure 4:**
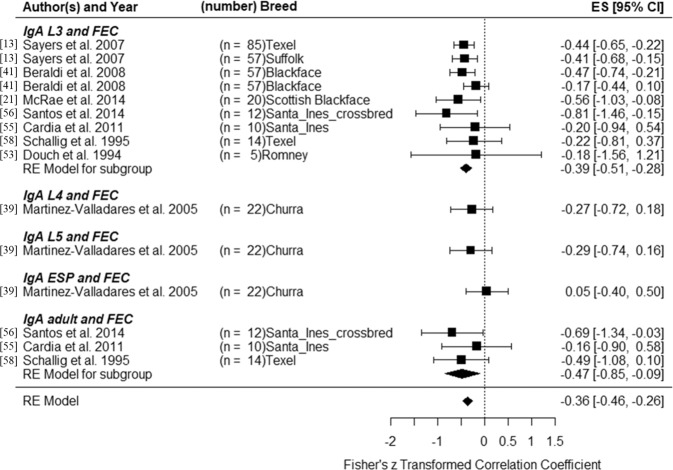
Forest plot for the correlation between IgA and fecal egg count (FEC). ES: effect size, CI: confidence interval.

In this paper, we have collected data from previous studies that measured correlations between FEC and blood IgA levels against different larval stages. We also calculated correlations using raw data for FEC and blood IgA level from these studies before re-analyzing all the data. Figure 4 shows the correlations between FEC and blood IgA levels against different larval stages from different studies. The overall correlation between IgA in blood and FEC was negative (*r* = −0.36, 95% CI = −0.46, −0.26). Correlations between FEC and blood IgA activity against L3 or adult were −0.39 (95% CI = −0.51, −0.28) and −0.47 (95% CI = −0.85, −0.09), respectively. Only one study measured the correlation between FEC and blood IgA activity against L4, L5 or ESP. Although IgA is produced locally at the mucosal surfaces and serum IgA is derived from the gastrointestinal tract^24^, few studies measured mucosal IgA, possibly because of the difficulties in sampling mucus. Serum IgA is easier to measure and highly correlated with mucosal IgA^40,41^. We were not able to re-analyze mucosal IgA. Although CarLA saliva IgA antibody test is currently being marketed (CARLA^®^ SALIVA TEST) as a powerful new tool for measuring parasite immunity in sheep (https://www.agresearch.co.nz/doing-business/products-and-services/carla-saliva-test/), no correlation was found between saliva IgA and serum or mucosal IgA^40^. However, antibodies in saliva may be binding directly to ingested L3.

### IgE response

The degranulation of mucosal mast cells is induced by the cross-linking of IgE on their surface. Following reinfection, a negative correlation between the concentrations of globule leucocytes (intraepithelial mast cells) and the *T. circumcincta* worm burden have been shown in sheep^48^. Indeed, the IgE antibody response mainly directed against L3 antigens, would be more prominent in previously infected sheep^49^.

In Romney sheep selected for almost two decades for high or low FEC following *T. colubriformis* infection, sheep from the low FEC line had higher total IgE (97 to 103%) and *T. colubriformis*-specific IgE (59 to 98%) compared to sheep from the high FEC line^50,51^. Similarly, after 7 weeks of grazing on a contaminated pasture, low FEC (based on the accumulated weekly measure) lambs (Greyface × Suffolk) had significantly higher systemic levels of IgE anti-HMW*Tc* (a major high molecular weight complex allergen from *T. circumcincta* L3) than high FEC lambs^52^. In addition, after 13 and 18 weeks on pasture, serum IgE anti-HMW*Tc* assays demonstrated an even greater difference between low and high FEC lambs. We conclude from these studies that high levels of IgE are associated with low levels of FEC in sheep. Pettit *et al*.^53^ showed that during the first and second year on pasture a higher concentration of blood circulating IgE-bearing cells was associated with a lower FEC of Scottish Blackface lambs.

Other studies comparing resistant and susceptible sheep breeds confirm the role of IgE as an important effector of the immune response to nematode infection in sheep^13,33^. Sayers *et al*.^13^ reported that the most notable difference of mucosal antibody isotype when comparing Texel (GIN resistant breed) and Suffolk (GIN susceptible breed) is IgE, after natural GIN infection. Mucosal IgE in Texel was significantly negative correlated with FEC (*r* = −0.48) and abomasum worm burden (*r* = −0.47). Also, the total IgE content in 5-6 month old Gulf Coast Native lambs increased significantly and was highest 7-14 days after artificial infections with *H. contortus* compared to the level in Suffolk lambs, which was confirmed in a natural infection experiment with the highest level of IgE at 14-42 days post infection^33^.

### IgG response

A review of studies investigating IgG and FEC after parasite infection in different sheep breeds^12,34,38,54^ suggested that IgG (especially IgG1) increases following infection and is associated with increased resistance to some GIN infections in sheep. Romney lambs had elevated levels of antibodies to *T. colubriformis* L3 excretory/secretory antigens which consisted predominantly of IgG1, reaching peak levels between days 42 and 77 post infection^54^. Bisset *et al*. (1996) found that the *T. colubriformis*-specific IgG1 response against both L3 and adult secretory/excretory antigens, was significantly higher in Romney lambs bred for low FEC compared to those selected for high FEC. IgG1 was negatively correlated with FEC (*r* = −0.60 and −0.48) and strongyle burden (*r* = −0.53 and −0.57) for both L3 and adult worm secretory/excretory antigens^38^.

Barbados Blackbelly lambs, which are resistant to *H. contortus*, had higher amounts of IgG anti-L3 between weeks 9 and 15 after infection. On the other hand, in the 9th week, sheep from the less resistant Columbia breed had lower IgG levels compared to the uninfected group^34^. An increase in IgG anti-L3 and IgG anti-adult *H. contortus* was also observed in Santa Ines ewes (a resistant breed) and its cross with Dorper, Ile de France, Suffolk and Texel, as a consequence of exposure to GIN larvae on pasture^55^. Santa Ines lambs had, after artificial infection with *T. colubriformis*, significantly higher specific serum levels of IgG anti-L3 and IgG anti-adult than the uninfected control group from week 4 (*P* < 0.05) to 13 (*P* < 0.01) post-infection^56^. A significant negative correlation was reported between *H. contortus* burden and IgG against L3 (*r* = −0.72), IgA against L5 (*r* = −0.61) and mast cells (*r* = −0.73) in Santa Ines male lambs, but not in Ile de France, which may be the reason that Santa Ines sheep had a lower FEC and worm burden than Ile de France sheep^12^. In a serial infection trial with Santa Ines crossbred lambs, *H. placei* infection induced high levels of IgG anti-L3 and IgG anti-adult compared with a control group, while animals serially infected with *H. contortus* induced high levels of IgG anti-adult but not IgG anti-L3 compared with the uninfected control group^57^.

### IgM response

IgM has an important role in the immune response as it represents the first class of antibodies produced following the initial exposure to a foreign antigen^58^. However, it is not normally present in the gastrointestinal mucus^59^. Only a few studies have investigated the IgM response in sheep during GIN infection and the findings from two studies did not suggest a major effect of IgM in sheep^33,38^. In selected Romney ram progeny, bred for genetic divergence in FEC, the correlations between IgM and both FEC or GIN burden were weak following an extended period of exposure to naturally contaminated pasture^38^. IgM did not differ between Gulf Coast Native (a resistant breed) and Suffolk lambs (a susceptible breed) in both artificial infections with *H. contortus* and natural infection experiments^25^.

In contrast, GIN challenge in Romney lambs gave rise to elevated levels of IgM anti-L3 after artificial infection with *T. colubriformis* L3^54^. A moderate increase of IgM serum antibody levels against both larval and adult antigens was found in Texel lambs in both primary infections and challenge infections with *H. contortus*^60^. Jejunal IgM anti-L3, after *T. colubriformis* infection, was the highest in resistant Merino animals with a tertiary infection, whereas susceptible animals with one infection had the lowest titres. This difference was not observed for abomasal IgM anti-L3 after *H. contortus* infection^61^.

### Immunoglobulin response in goats

The strategies developed by goats appear to be different from those observed in sheep, to regulate GIN infections^62^ and to establish immunity^63^, but only few studies have investigated the immune response against GIN in goats. Bambou *et al*.^64^ found that serum antibodies IgA anti-L3, IgE anti-L3 and IgA anti-ESP, IgE anti-ESP increased significantly after L3 *H. contortus* infection in both susceptible and resistant 11 months old Creole kids. At the same time IgG anti-L3 and IgG anti-ESP levels were weak in both groups. Similarly, McBean *et al*.^23^ reported no consistent differences in IgA, IgE or IgG levels between Scottish Cashmere goats selected for low FEC (generations F2 to F9) and control lines (unselected) after artificial infection with *T. circumcincta* and during the grazing season. On the other hand, IgA anti-ESP, IgA anti-L3 and IgE anti-L3 were genetically correlated with FEC (0.84 ± 0.13, 0.72 ± 0.18 and −0.32 ± 0.08, respectively), while they did not find any phenotypic correlation between them^65^. A phenotypic correlation is the correlation between records of two traits on the same animal. Genetic correlation, traditionally calculated from pedigree data, is a measure of the genetic relationship between two traits^66^. A high genetic correlation between two traits is generally supported by genes that are usually co-inherited. The phenotypic correlation estimate the correlation between two traits and is depend both on additive genetic and of environmental effects^66^. Recently, it has been suggested that the lack of functionality of the immune response mediated by IgA and eosinophil against natural nematode infection in Boer goats would be due to a dysfunctional transmembrane domain of the high affinity IgA receptor^67^. Altogether these results indicate that the humoral response against GIN infection is less effective in goats than sheep and does not probably play a major role in resistance to nematode infections in goats.

### Genomic studies on GIN resistance

Identifying genetic markers of resistance and/or susceptibility could improve the efficiency of breeding programs. The first approach used to search for genetic markers associated with host resistance was a study of associations between variants of genes related to the host immune response and phenotypic resistant traits^68^. The genetic markers that have been most frequently associated with nematode resistant are those from the major histocompatibility complex (MHC) region on *Ovis aries* chromosome 20^69–74^. MHC genes are highly polymorphic^69,74^ and play important roles in presenting processed antigens to host T lymphocytes, causing T cell activation. The second most frequently region identified in studies for resistance to GIN infection is the interferon ɣ (IFNG) gene on *O. aries* chromosome 3^75,76^. The majority of studies of quantitative trait loci (QTL) focused on the association/ linkage between genomic regions and FEC. Only few studies examined the association between genomic regions and immunoglobulins level during GIN infection. Table 2 shows the genomic regions that were reported to be linked or associated with immunoglobulin-mediated resistance to GIN infection. Similarly to phenotypic studies, the majority of genomic studies focussed on the IgA response during GIN infection^73,75,77–79^, two studies considered the IgG response^80,81^ and only one study examined the IgE response^80^.

**Table 2:** Genomic regions associated with immunoglobulin-resistance to GIN infection.

Chr^a^	Breed	Infection, parasite species	Association	Marker intervals (MI)/SNP/candidate gene (CG)/allelic effects	Reference
0	Scottish Blackface	Natural, mainly T. circumcincta	IgA (L3)	SNP: s27388.1	^78^
1	Churra	Strongylidae	IgA	OAR1 markers at position 37 cM (ILSTS044)	^106^
1	Spanish Churra	Natural, mainly T. circumcincta	IgA (L4)	MI: BMS835 - ILSTS044	^77^
3	Soay	Natural, mainly T. circumcincta	IgA (L4)	CG: (o(IFN)-γ)*126 allele	^107^
3	Scottish Blackface	Natural, mainly T. circumcincta	IgA (L3)	MI: KD103 - LYZ	^73^
3	Scottish Blackface	Natural, mainly T. circumcincta	IgA (L3)	SNP: OAR3_227580261.1	^78^
3	Scottish Blackface	Natural, mainly T. circumcincta	IgA (L3)	SNP: OAR3_215619424.1	^78^
4	Scottish Blackface	Natural, mainly T. circumcincta	IgA (L3)	SNP: OAR4_90051359.1	^78^
4	Scottish Blackface	Natural, mainly T. circumcincta	IgA (L3)	SNP: OAR4_87762617.1	^78^
4	Scottish Blackface	Natural, mainly T. circumcincta	IgA (L3)	SNP: s57016.1	^78^
5	Romane × Martinique Black Belly backcross	Artificial, H. contortus	IgG (ESP)	MI: OAR5_67605574.1–OAR5_67883800_X.1	^81^
5	Romane × Martinique Black Belly backcross	Artificial, H. contortus	IgG (ESP)	MI: OAR5_100699982.1–DU183841_402.1	^81^
6	Scottish Blackface	Natural, mainly T. circumcincta	IgA (L3)	SNP: OAR6_107079726.1	^78^
8	Churra	Natural, mainly T. circumcincta	IgA (L4)	SNP: OAR8_53084022.1	^79^
8	Churra	Natural, mainly T. circumcincta	IgA (L4)	SNP: s42819.1	^79^
9	Churra	Strongylidae	IgA	OAR9 markers at 50 cM position	^106^
9	Romane × Martinique Black Belly backcross	Artificial, H. contortus	IgG (ESP)	MI: OAR9_85325486.1–s48117.1	^81^
10	Churra	Natural, mainly T. circumcincta	IgA (L4)	SNP: s56461.1	^79^
10	Churra	Natural, mainly T. circumcincta	IgA (L4)	SNP: OAR10_23921485.1	^79^
10	Churra	Natural, mainly T. circumcincta	IgA (L4)	SNP: s61799.1	^79^
11	Churra	Natural, mainly T. circumcincta	IgA (L4)	SNP: DU232778_232.1	^79^
12	Churra	Natural, mainly T. circumcincta	IgA (L4)	SNP: s68938.1	^79^
14	Romane × Martinique Black Belly backcross	Artificial, H. contortus	IgG (ESP)	MI: OAR14_48832510.1–s30682.1	^81^
14	Churra	Natural, mainly T. circumcincta	IgA (L4)	SNP: OAR14_21336208.1	^79^
15	Churra	Natural, mainly T. circumcincta	IgA (L4)	SNP: s75729.1	^79^
20	Scottish Blackface	Natural, mainly T. circumcincta	IgA (L3)	MI: BM1815 - DRB1	^73^
20	Rhönschaf	Artificial, H. contortus	IgL	DYMS1 (DYA) allele C	^71^
20	Scottish Blackface	Natural, mainly T. circumcincta	IgA (L3)	SNP: OAR20_40924783_X.1	^78^
21	Romane × Martinique Black Belly backcross	Artificial, H. contortus	IgG (ESP)	MI: s27845.1–OAR21_14592163.1	^81^
23	Romney × Coopworth	Natural, mainly T.colubriformis	IgE (total)	MI: Centomere - BM226	^80^
23	Romney × Coopworth	Natural, mainly T.colubriformis	IgG (L3)	MI: McMA1 - ADCYCAP1	^80^
25	Churra	Natural, mainly T. circumcincta	IgA (L4)	SNP: s21640.1	^79^
26	Romane × Martinique Black Belly backcross	Artificial, H. contortus	IgG (ESP)	MI: OAR26_21857857.1–OAR26_22456940.1	^81^

^a^Chromosome.

### Factors that impact animal response to GIN infection

A number of factors impact the immune response to parasite infection. We focus here on the factors of the animal itself, such as genetic differences, age of the animal and immunological experience, infection period, or type of infection (i.e. natural or experimental).

### Age of the animal and immunological experience

Our review on immune responses to parasite infection in sheep suggests that the age of the animal influences the immune response to GIN infection. Even though the traits investigated were different, three studies have identified age-related differences in the immune response^82–84^. High eosinophil counts were significantly correlated with low FEC in naturally infected with *T. circumcincta* Scottish Blackface lambs which were at least 3 months of age, where the correlations were −0.33, −0.14 and −0.24 after 3, 4 and 5 months of age, respectively^83^. Also the correlations between IgE activity and FEC in Texel lambs following 4 weeks of natural mixed nematode infection were only significant at 5 and 6 months, while it was not significant at 7 months of age when natural exposure would be declining due to the onset of late autumn and winter^82^. We found that many studies discussed their results without taking the age structure of the chosen experimental cohort as an important factor into account. However, from the studies using age different cohorts, we conclude that differences in immune reactivity might be partly explained by age.

Immunological experience has a huge impact on the response to parasite infection and is partly confounded with animal age. Immune response was reported to be low and delayed in primary-infected lambs while it is higher and rapid in previously-infected animals^60,85,86^. The majority of the studies (85%, n = 35) focus on lamb immune response as model for nematode infection and half of these studies (n = 18) used non-naïve lambs. It has been shown that genetic variation in resistance to GIN is associated with the development of an acquired immune response, which explains why the pathophysiological impact of these parasitic infections is more important in growing lambs compared to mature sheep^87^. Also, in lambs genetic variation in FEC is not correlated with genetic variation in the total number of worms but rather to female worms length and consequently their fecundity^88^. In contrast to lambs, mature sheep may have the ability to limit both fecundity and worm numbers^87^. Hence the immune mechanisms could differ.

### Infection period

Days post infection (d.p.i) represents also a very important factor that may affect the results obtained. The majority of the research measured host immune response within 0 to 6 weeks post infection, while other studies indicate that they did not find significant immune response until about 9 weeks post infection^34,37,42^.

IgG level at 60 d.p.i was significantly correlated with worm burden (*r* = 0.235 to 0.247) and total antibody level was significantly correlated with worm length (*r* = 0.316) and FEC (*r* = −0.148), whereas the correlations were not significant at 30 d.p.i in Merinoland sheep infected with *H. contortus*^37^. A similar difference was also observed in Rhön sheep, the total antibody value was significantly correlated with worm burden (*r* = 0.372 to 0.378) at 60 d.p.i but not at 30 d.p.i^37^. Similarly, in Blackface lambs exposed continuously to infection of *T. circumcincta*, anti-*T. circumcincta* IgA levels were inversely correlated with FEC and increased with time from *r* = −0.17 (NS) at 14 d.p.i to *r* = −0.44 at 84 d.p.i (*P* < 0.001)^42^. Moreover, the difference of the anti-*T. circumcincta* IgA level between resistant and susceptible sheep diverged with time and was significant from 56 d.p.i. onwards^42^.

Comparing immune response to GIN in resistant and susceptible sheep breeds, sheep of both Barbados Blackbelly (resistant) and Columbia (susceptible) breeds showed a significant increase in blood eosinophils from 1 to 9 weeks post infection, these levels decreased suddenly thereafter in Columbia lambs^34^. A similar pattern was observed in the same study for IgG anti-L3 levels, Barbados Blackbelly lambs had a higher amount of IgG anti-L3 between weeks 9 and 15 after infection with a positive significant regression (0.79), while the regression in Columbian lambs was negative and not significant (−0.59) at week 9. These results suggest that at the beginning of the infection the immune response may not differ a lot between resistant and susceptible animals but differences become more apparent 9 weeks after infection.

An interlaced issue is the effect of age and infection period, significantly greater nematode-specific serum antibody activities were reported in Texel compared to Suffolk sheep for all isotypes (IgG1, IgG2, IgA and IgE) at 14 and 17 weeks of age with increasing divergence between the breeds as age increased^13^. Meanwhile these differences could be explained as the effect of infection period as in this experiment the lambs were exposed to natural infection after staying on pasture within 1 to 3 days of birth, so these differences in response could be due to age or infection period or a combination of both.

### Infection type (natural/artificial)

The majority of the studies measuring at least one immunoglobulin as a response to GIN infection used single infections (n = 25, 59.5%) with high number of GIN larvae or natural infections (n = 10, 24%). A natural infection occurs gradually and results from large single infections may not reflect the pattern of a natural infection^34^. However, in a natural infection, we cannot control infection dose or determine the exact time of infection; consequently the results may not accurately reflect the difference between susceptible and resistant animals. A suggested solution is to use artificial infection of different doses with a low number of larvae weekly (trickle infection) with which infection dose, the time of infection and infection specificity are controlled^34^. We only found 12% (n = 5) of the studies used trickle infection for their experimental design when measuring immunoglobulin response to GIN infection. The only study that measured the immune response to GIN infection under single and trickle infection found that mean optical densities for serum IgG in naïve lambs (resistant) was significantly higher than in Suffolk lambs (susceptible) in single infection groups at 14 and 21 d.p.i with no significant difference between these breeds in trickle infection groups at same time points^33^.

### Summary for immunoglobulins role against GIN infection

Results from published studies, which have reported significant correlations between different immunoglobulins and parasite parameters (FEC, worm burden and worm length) are summarized in Table 3. These studies suggest a role for L3 antigen-specific IgA, IgE and IgG responses in resistance to GIN in sheep. Also the role of L4 antigen-specific IgA for the resistance to GIN in sheep appears more important than the role of IgA against L3.

**Table 3:** Immunoglobulin classes reported to be significantly correlated with resistance traits.

Ig class	Larval stage	Parasitological parameter	Breed	State	Inf.^a^, GIN sp.^b^	Reference
AB	L3, adult	WormBurden	Romney	non-native	NI, *H. contortus*	^38^
		FEC	Merinoland	native	AI, *H. contortus*	^37^
		WormBurden	Rhön	native	AI, *H. contortus*	^37^
		WormLength	Merinoland	native	AI, *H. contortus*	^37^
IgA	L3	FEC	Blackface	non-native	AI, *T. circumcincta*	^42^
			Texel, Suffolk	native	NI, *T. circumcincta*	^13^
			Romney*Texel* FinnishLandrace	non-native	NI, *H. contortus*	^45^
			Pelibuey	native	NI, *H. contortus*	^108,109^
		WormBurden	Blackface	non-native	AI, *T. circumcincta*	^42^
			Texel, Suffolk	native	NI, *T. circumcincta*	^13^
		WormBurden, inhibited L4	Scotch Mule (Blackface ewe * Blue-faced Leicester ram)	native&non	AI, *T. circumcincta*	^39^
	L4	FEC	Churra	non-native	AI, *T. circumcincta*	^40^
		WormLength	Scottish Blackface	non-native	AI, *T. circumcincta*	^41^
			Churra	non-native	AI, *T. circumcincta*	^40^
	L5	FEC	Churra	non-native	AI, *T. circumcincta*	^40^
		WormBurden	Santa Ines	non-native	NI, *H. contortus*	^12^
IgE	L3	FEC	Texel	non-native	NI, *T. circumcincta*	^82^
			Texel	native	NI, *T. circumcincta*	^13^
		WormBurden	Texel, Suffolk	native	NI, *T. circumcincta*	^13^
IgG	L3	FEC	Blackbelly and Columbia	native	AI, *H. contortus*	^34^
		WormBurden	Merinoland	native	AI, *H. contortus*	^37^
			Santa Ines	non-native	NI, *H. contortus*	^12^
	L3, adult	WormBurden, L4Burden, AdultBurden	Merino	non-native	AI, *H. contortus*	^97^
IgG1	L3	FEC	Texel, Suffolk	native	NI, *T. circumcincta*	^13^
			Romney*Texel* FinnishLandrace	non-native	NI, *H. contortus*	^45^
		WormBurden	Romney	non-native	NI, *H. contortus*	^38^
IgG2	L3	FEC, WormBurden	Texel, Suffolk	native	NI, *T. circumcincta*	^13^

^a^Inf.: Infection protocol, artificial infection (AI), natural infection (NI).

^b^GIN sp.: Gastrointestinal nematode species.

**Figure 5:**
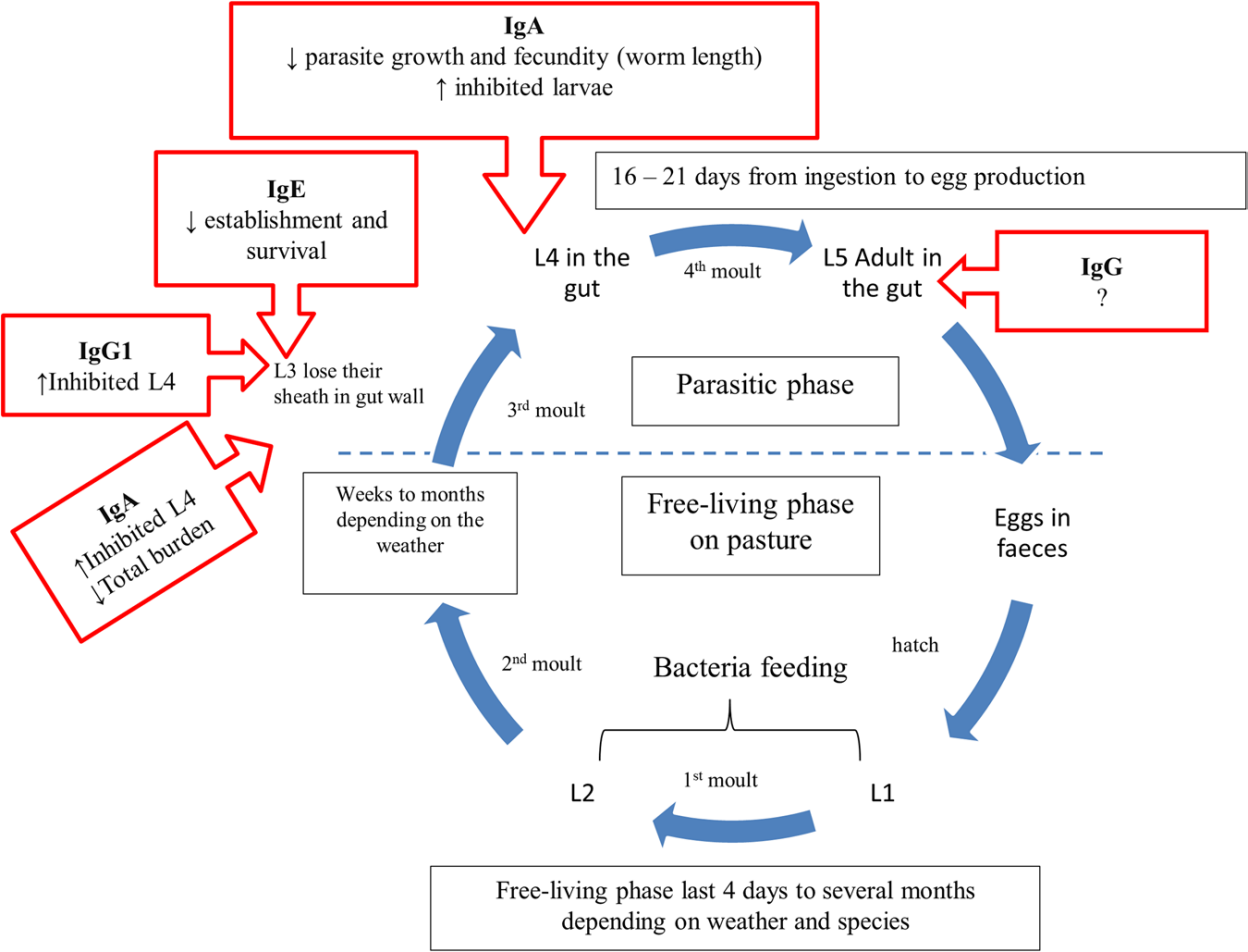
Immunoglobulins suggested role in resistant to gastrointestinal nematode during its life cycle.

Three major mechanisms of immunity to nematodes have been described in sheep, regulation of the establishment rate of infective larvae, suppressed nematode growth and thus fecundity, and the expulsion of adult worms; a combination of these mechanisms is possible^24^. Figure 5 shows the gastrointestinal nematode life cycle and summarizes the suggested role of different immunoglobulins in the three major mechanisms of immunity to nematodes. Reduced parasite establishment and survival is associated with IgE activity mainly against incoming third stage larvae (L3) in concert with mast cells as cross-linking of IgE on the mast cell surface leading to mast cell degranulation^82,89^ with more prominent response in previously infected animals^49^. Reduced parasite growth and fecundity is correlated with increased local IgA activity against fourth stage larvae^59,89,90^. Increased number of inhibited larvae is correlated with IgG1 activity against the third stage larvae^54,60^ beside IgA activity against the third and fourth stage larvae^89,90^.

## Conclusions

The selection of animals with a high immune response to GIN infection is a promising method of reducing the negative impact of these infections on grazing small ruminants. We have highlighted factors that differ across studies and affect the immune response to GIN infection. Indeed, it is essential that future studies take into account and mention the age of the animals, the infection experience and the type of infection (i.e. single vs trickle). One another important point is the need to normalize the measurements of immunoglobulin concentrations. The use of different units and/or optical density to measure immunoglobulin levels at different time post-infection produces results that are incomparable between studies. One way to overcome this issue is to use the change ratio between day 0 or an uninfected group and different time post-infection or to develop methods for the quantification of the immunoglobulins with standard curves. This effort for standardization should potentially allow to take advantage of the research results produced by implementing breeding programs for higher resistance to GIN infection.

## Material and Methods

The study methodology followed the guideline of “Preferred Reporting Items for Systematic Reviews and Meta-Analyses: The PRISMA Statement”^91^. The literature search had been conducted using electronic databases. Web of Science and PubMed were used as databases to cover the literature on small ruminants and parasites. Besides, the reference lists from five literature reviews, all published after 2008, were additionally searched^24,28,29,89,92^. Studies were closely evaluated and selected for inclusion if they measured at least one immunoglobulin type and the data were extractable. The literature review revealed 41 studies which measured at least one immunoglobulin: 35 focused on lamb immune response (18 used non-naïve lambs) and 7 on yearlings. Overall, the methodological quality of included studies was evaluated qualitatively with an 9-point scoring system based on SYRCLE’s risk of bias tool^93^: (1) Statement on ethical approval or guidelines followed for using animals in the study clearly mentioned, (2) Adequate allocation sequence generation, (3) similar groups at baseline, (4) Allocation concealment, (5) Random housing, (6) Random outcome assessment, (7) Outcome assessment blinded and (8) incomplete outcome adequately addressed (Fig. 1).

Information extracted from each study included nematode genus, host (sheep or goat), infected breed, host classification (resistant or susceptible), host sex, host age (months), age at weaning (weeks), infection period (days post infection), sample tissue, infection type (artificial or natural), number of animals per group, immunological state (naïve or non-naïve), total number of infected larvae, larval details and infection method for artificial infection (single or trickle). Measured traits of interest included both parasitological and immunological parameters. If data were provided in graphical form, traits means were extracted using WebPlotDigitizer (version 3.8)^93^. These summary measures beside the study information previously mentioned were entered into an electronic spreadsheet in Microsoft Excel and a dataset was built. The Metafor package in R (version 3.5.1) was used to analyze the correlation coefficient between FEC and immunoglobulins. A REML model was used in which the effect size was calculated according to the number of animals used in each study with 95% confidence interval. The possibility of publication bias across studies reporting correlation coefficients was assessed by a visual analysis of a funnel plot of the standard error by the Fischer’s Z transformed correlation coefficients and formally by using Egger weighted regression test (Metafor package in R version 3.5.1).

## Supplementary information

Supplementary information
